# Manufacturing of Microstructural, Mechanical, and Corrosion Properties of MnAlCuFeTi High-Entropy Nanomaterials: Influence of Mechanical Alloying Time and Sintering Temperature

**DOI:** 10.3390/nano16070401

**Published:** 2026-03-26

**Authors:** Seyit Çağlar, Cengiz Temiz

**Affiliations:** 1 Department of Metallurgical and Materials Engineering, Zonguldak Bülent Ecevit University, Zonguldak 67100, Türkiye; 2 Department of Electronics and Automation, Alaplı Vocational School, Zonguldak Bülent Ecevit University, Zonguldak 67850, Türkiye; cengiztemiz@beun.edu.tr

**Keywords:** MnAlCuFeTi, high entropy alloy, nanomaterials, mechanical alloying, corrosion

## Abstract

This study explores how variations in mechanical alloying time and sintering temperature influence the microstructure, mechanical properties, and corrosion resistance of MnAlCuFeTi high-entropy alloys (HEAs). The MnAlCuFeTi alloy was produced by means of mechanical alloying for 5, 10, 15, and 20 h. Afterward, the alloy samples were sintered at two different temperatures: 550 °C and 650 °C. Structural properties were analyzed using X-ray diffraction (XRD), scanning electron microscopy (SEM), and energy-dispersive X-ray spectroscopy (EDX). Analysis of grain sizes, calculated using the Scherrer formula from SEM images, confirmed that grain size had decreased to the nanostructured regime and that microstructural homogeneity had improved. Corrosion behavior was evaluated using polarization curves, corrosion current density (*I_corr_*), and corrosion rate measurements. The results show that increasing the mechanical alloying time reduces the alloy’s grain size, thereby improving its mechanical and corrosion resistance. At a sintering temperature of 550 °C, *I_corr_* and corrosion rate decrease with increasing grinding time, whereas at 650 °C, although high temperatures accelerate diffusion processes and increase phase homogeneity, they weaken corrosion resistance. These findings emphasize the importance of balancing alloying time and sintering temperature to optimize performance in high-corrosion-resistant HEA applications.

## 1. Introduction

Today, nanomaterial production should focus not only on achieving high mechanical performance but also on minimizing environmental impacts, optimizing production processes for energy efficiency, and reducing dependence on critical raw materials [1,2,3]. High-entropy alloys (HEAs) have emerged as a significant innovation in materials science. These alloys are composed of at least five principal elements, each present in nearly equal atomic proportions. This unique composition leads to the formation of simple crystal structures, such as Face-Centered Cubic (FCC) or Body-Centered Cubic (BCC), driven by the high configurational entropy effect [4,5,6].

Traditional alloy systems, designed around a primary element, have now been expanded by HEAs, which introduce a new approach to multi-component systems [7]. This new approach provides a broader range of compositions and material properties, enabling the system to meet diverse engineering performance requirements. HEAs are characterized by exceptional properties, including high strength, excellent wear and corrosion resistance, enhanced thermal stability, and remarkable structural integrity. These attributes make them ideal for industries where long service life and high reliability are crucial, such as defense, energy systems, automotive, marine, and various industrial applications [8,9,10].

In terms of sustainability, HEAs offer significant advantages by combining high mechanical performance with environmental benefits, thanks to the versatility of their constituent elements [11,12]. Unlike traditional alloys that contain cobalt, rare-earth elements, or other strategically critical metals, HEAs are made from abundant, non-toxic, and recyclable materials like Mn, Cu, Fe, Ti, and Al [13,14]. This makes them a more environmentally and economically sustainable choice. Moreover, the superior hardness, wear resistance, corrosion resistance, and extended service life of HEAs help to minimize the need for frequent material replacements, reduce maintenance demands, and lower energy consumption throughout their lifecycle [15,16,17].

Powder metallurgy techniques enable industrial production of nanocrystalline materials by consolidating and sintering powders produced via high- or medium-energy ball milling or mechanical alloying (MA) [18]. MA minimizes element losses, reduces energy consumption, and facilitates homogeneous alloy formation at lower processing temperatures. It promotes fine-grained, homogeneous microstructures, improving hardness, wear resistance, and corrosion resistance. Compared to traditional melting and casting methods, MA is a more energy-efficient and environmentally friendly production route [19,20,21]

The sintering stage directly affects HEA consolidation, phase stability, and microstructural evolution. Sintering temperature controls atomic diffusion rates, grain growth, and solid-solution stabilization. While advanced sintering techniques require high energy and incur higher equipment costs, traditional sintering methods offer lower energy consumption when optimized for temperature and time, enabling a more cost-effective and environmentally friendly production approach [22,23].

The primary aim of this study is to fabricate a MnAlCuFeTi-based high-entropy alloy (HEA) using the mechanical alloying method and to systematically assess how different grinding times (5, 10, 15, and 20 h) and sintering temperatures (550 °C and 650 °C) influence phase composition, microstructure, density, mechanical properties, wear resistance, and corrosion behavior. The selected alloy system, free of critical elements and composed of low-cost, non-toxic materials, is an excellent candidate for sustainable engineering applications. While existing research on MnAlCuFeTi and similar HEAs typically focuses on advanced sintering methods or examines individual properties (such as hardness or microstructure), this study fills a critical gap by simultaneously optimizing mechanical alloying time and sintering temperature and exploring the interrelationships among multiple properties within a single production process.

## 2. Experimental Procedures

### 2.1. Preparation of the High-Entropy Alloy MnAlCuFeTi

In this study, high-entropy alloys (HEAs) based on MnAlCuFeTi were synthesized using commercially available pure powders: Mn (99.5%), Al (99.9%), Cu (99.9%), Fe (99.9%), and Ti (99.5%), sourced from Nanografi Nano Technology Inc. (Ankara, Türkiye). The powders had an average particle size in the micrometer range. The alloy composition was designed to ensure equal atomic ratios of each element, and the mixing ratios were optimized to achieve this composition. The mechanical alloying process was conducted in a planetary ball mill (Fritsch Pulverisette 5, Idar-Oberstein, Germany) with a 250 mL hardened stainless steel grinding bowl and 10 mm hardened stainless steel balls. The ball-to-powder weight ratio was maintained at 10:1 throughout the milling process. The effective diameter of the main disk was 250 mm. Stearic acid was used as a process control agent (PCA) at 1.5 wt.% to reduce powder adhesion to the milling chamber walls and enhance powder flowability. The milling was carried out in an argon atmosphere at room temperature with a rotational speed of 250 rpm. Milling cycles were set to 30 min of continuous milling followed by 30 min of rest to prevent excessive temperature rise. To study the effect of varying processing parameters, milling times of 5, 10, 15, and 20 h were tested. These conditions were selected to evaluate the influence of alloying time on the microstructure, mechanical properties, and corrosion resistance of the synthesized MnAlCuFeTi high-entropy alloys.

### 2.2. Compaction and Sintering

The HEA powders, after mechanical alloying, were compacted into cylindrical pellets with a diameter of 13 mm using a single-axis cold-pressing method at 650 MPa for 1 min. These pressed pellets were then sintered in a Protherm vacuum furnace (Alser Teknik, Ankara, Türkiye) under an argon atmosphere at temperatures of 550 °C and 650 °C for 2 h each. The selected sintering temperatures were carefully chosen to promote adequate diffusion while minimizing excessive grain growth. Following the sintering process, the samples underwent progressive polishing, starting with 600-grit and progressing to 4000-grit. Final polishing was carried out using a 1-micron cloth along with a polishing solution. To conclude the preparation, the samples were ultrasonically cleaned to remove any residual contaminants.

### 2.3. Density Measurements

The experimental densities of the sintered MnAlCuFeTi high-entropy alloy pellets were determined by averaging five measurements using the WSA-224 density measurement device (Weightlab, Ankara, Türkiye), which operates on Archimedes’ principle, in accordance with ASTM D792. The theoretical density of the alloy was calculated using Equation (4), which incorporates the densities of the individual constituent elements.

### 2.4. Structural and Microstructural Characterization

Phase analyses of the MnAlCuFeTi-based high-entropy alloys were conducted using a RIGAKU SmartLab X-ray diffractometer (Rigaku Corporation, Tokyo, Japan). X-ray diffraction (XRD) measurements were performed using Cu Kα radiation, enabling the determination of the phase composition and crystal structures of the samples from their diffraction patterns. The surface morphology and microstructural characteristics of the samples were investigated using a TESCAN MAIA3 XMU analytical scanning electron microscope (SEM) (TESCAN Brno s.r.o., Brno, Czech Republic). The MAIA3 XMU system provides high resolution at low acceleration voltages, ensuring accurate microstructural observations without causing surface damage or charge accumulation, even for non-conductive or sensitive materials. In the SEM analyses, the grain structure, porosity, and microstructural homogeneity were examined at different magnifications. The elemental distribution and chemical composition of the alloys were analyzed through energy-dispersive X-ray spectroscopy (EDX) integrated into the SEM system. EDX analysis and elemental mapping were employed to assess elemental distribution within the alloy and identify potential regions of segregation.

### 2.5. Microhardness and Wear Testing

Vickers microhardness measurements were conducted using a Shimadzu HMV-G21 device (Shimadzu Corp., Kyoto, Japan) with a 100 g load and a 15-s dwell time. Ten measurements were taken from different regions of each sample, and the average value was calculated. Wear tests were carried out using a TRIBOtechnic-TRIBOtester device (TRIBOtechnic, Clichy, France) under a load of 8 N, a total sliding distance of 100 m, and a sliding speed of 12 m/s. The cross-sectional areas of the wear tracks were measured using a Taylor Hobson 2D profilometer (Leicester, UK). The tests were conducted at a relative humidity of 32–38% and a temperature range of 23–28 °C. Wear volume (Equation (1)) and wear rate (Equation (2)) were determined from the wear-track data obtained [24].

(1) V=A.l

(2) $$WR=\frac{V}{S}$$

where:

*V*: Wear volume (mm^3^), *S*: Sliding distance (m);
*A*: Area of the worn path (mm^2^), *l*: Wear Length (mm);
*WR*: Wear Rate (mm^3^/m).

### 2.6. Electrochemical Testing

**Figure 1:**
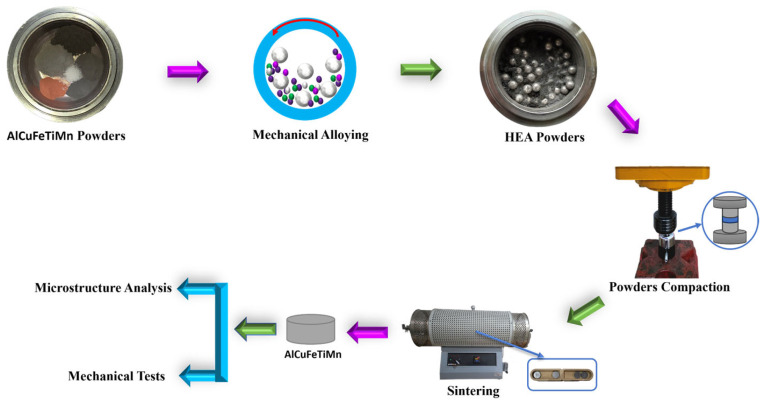
Schematic illustration of the production and characterization process of a high-entropy MnAlCuFeTi alloy.

Electrochemical tests were performed using a Gamry Reference 1010E potentiostat (Gamry Instruments, Warminster, PA, USA) at 24 ± 2 °C in a 3.5% NaCl solution. The corrosion current density (*I_corr_*) was determined using the Tafel extrapolation method, and the corrosion rate was subsequently calculated from it. During the calculations, the theoretical alloy compositions, mixing rules, and relevant weight percentages were taken into account. Each stage of the test process, from sample preparation to application, is clarified in the schematic flow diagram shown in Figure 1. This visual approach enhances clarity of the experimental methodology and ensures test reproducibility. The corrosion rate (Equation (3)) was calculated using the following formula [24]:

(3) $$CR=k\frac{i_{corr\;}EW}{\rho }$$

where:

*k* = 3.27 × 10^−3^, constant, *I_corr_* = corrosion current density (µA/cm^2^), *EW* = equivalent weight (g.eq^−1^), and *ρ* = experimental density (g.cm^−3^).

## 3. Results and Discussion

The experimental densities and their respective relative densities of the MnAlCuFeTi high-entropy alloy, sintered at 550 °C and 650 °C, are shown in Table 1 and Table 2, respectively. For both temperatures, the theoretical density value was determined to be 5.16 g/cm^3^. Experimental densities were measured according to different mechanical alloying times (5 h, 10 h, 15 h, and 20 h), and relative densities were calculated for each sample. An increase in experimental density and, consequently, relative density values was observed as the sintering time increased. The relative density of samples sintered at 550 °C ranged from 83.6% to 89.2%, while for samples sintered at 650 °C, these values ranged from 84.3% to 89.8%. These findings suggest that both sintering temperature and duration enhance the alloy’s density and structural integrity. The theoretical density, as calculated using Equation (4), is the weighted average of the constituent elements’ densities. The relative density was calculated using Equation (5), the ratio of the experimental to the theoretical density [24].

(4) $$\rho_{theo\;}=\frac{1}{\;\sum_{i=1}^{n}\frac{\omega_{i}}{\rho_{i}}\;}\;\;$$

(5) $$\rho_{rel}(\%)=\left(\frac{\rho_{exp}}{\rho_{theo}}\right)\times 100$$

### 3.1. XRD Analysis

**Figure 2:**
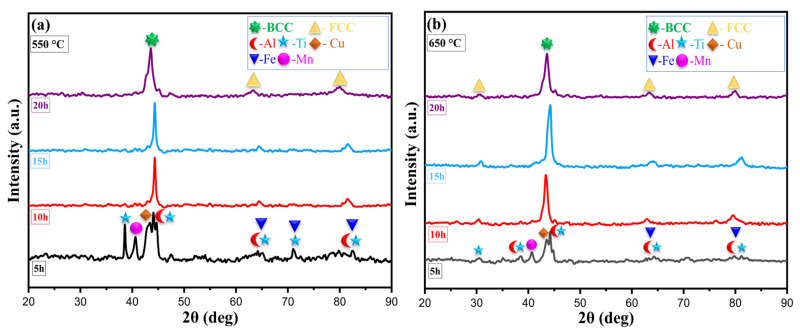
X-ray diffraction patterns of the high-entropy MnAlCuFeTi alloy sintered at (**a**) 550 °C and (**b**) 650 °C.

XRD analysis was conducted to investigate the crystal structures and phase composition of the MnAlCuFeTi high-entropy alloy, with the patterns shown in Figure 2 for samples sintered at 550 °C and 650 °C. Distinct peaks corresponding to BCC (Body-Centered Cubic) and FCC (Face-Centered Cubic) crystal structures were observed in the patterns [25]. In addition, phases of elements such as Cu, Ti, Fe, Mn, and Al became more pronounced as sintering temperature and grinding time varied. As the grinding time increased, more pronounced peaks appeared in the XRD patterns of the alloy sintered at 550 °C. At this sintering temperature, the effect of the Fe, Mn, and Al phases is more pronounced. This indicates that in samples sintered at 550 °C, there are more phase separations between the crystal structures of these elements, meaning that the elements cannot diffuse sufficiently, and a heterogeneous phase distribution emerges. It is understood that this process, which begins with a 5 h grinding period, yields better crystallization and a more homogeneous structure as the grinding time increases.

As expected, sharper peaks were observed in the XRD patterns at a sintering temperature of 650 °C. This indicates that the high sintering temperature accelerates atomic diffusion, improving crystallization and making the phases more stable. The effect of elements such as Fe, Mn, and Al became less pronounced in samples sintered at 650 °C. This indicates that at high temperatures, the elements are distributed more homogeneously and the crystal structures become more stable. Sintering at 650 °C minimized the phase separation observed at lower temperatures and ensured a more balanced elemental distribution within the alloy [26].

As a result, high sintering temperatures (650 °C) have led to the formation of a more stable crystal structure, which emerges in a more homogeneous phase due to high atomic diffusion rates. In samples sintered at 550 °C, however, the low temperature does not provide sufficient energy for the crystal lattices and phases to become fully stable. This innovation confirms that high temperatures and long grinding times are significant factors in crystallization and phase stabilization in high-entropy alloys.

### 3.2. SEM-EDX Analysis

**Figure 3:**
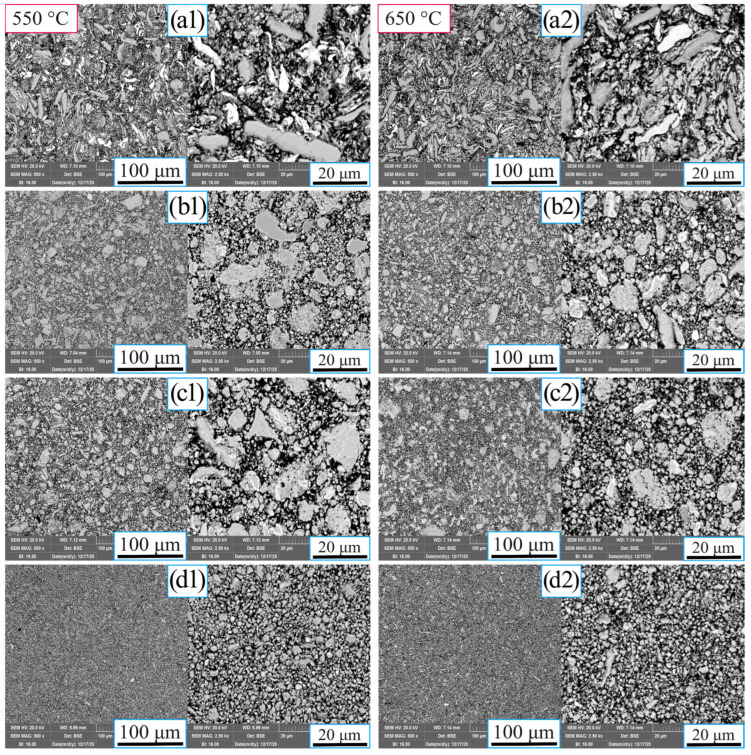
SEM micrographs of the high-entropy MnAlCuFeTi alloy sintered at 550 °C and 650 °C, shown at various magnification levels. Left column (550 °C): (**a1**) 5 h, (**b1**) 10 h, (**c1**) 15 h, (**d1**) 20 h, different magnifications. Right column (650 °C): (**a2**) 5 h, (**b2**) 10 h, (**c2**) 15 h, (**d2**) 20 h, different magnifications.

SEM (Scanning Electron Microscope) images of the sintered MnAlCuFeTi high-entropy alloy at sintering temperatures of 550 °C and 650 °C at different magnification levels are presented in Figure 3. At 550 °C during sintering (left column), in samples obtained after a 5 h grinding time, the grain size was on the micrometer scale, and the microstructure was not homogeneous. Furthermore, it is observed that elements such as Ti exhibit flat, sharp-edged structures, suggesting that the cold-working and fracture cycles have not been fully completed and, as a result, the crystal structures have not reached full maturity [27]. At grinding times of 10, 15, and 20 h, the grains fragmented, and the average became more homogeneous. During the 20 h grinding period, the particles were reduced to nanometric levels, and the micro-transfers became quite fine-grained.

Observations of samples prepared with the same grinding times as the sample sintered at 650 °C (right sample) reveal that a higher sintering temperature accelerates diffusion, enhancing particle reduction and leading to a more homogeneous structure. Images obtained after 5 h of grinding show that the particles are large, whereas those at 650 °C produce a more homogeneous structure. With a 10 h grinding time, the particles are smaller, and the structure is more homogeneous, with Ti and other elements forming smaller, spherical structures. At grinding times of 15 and 20 h, the particle temperature decreased to the nanometric range, and more homogeneous, spherical structures were observed in the microstructure. This indicates that longer grinding times make particle-size reduction more efficient and that spherical structures develop as grinding time increases.

As a result, sintering at 550 °C showed that irregular particle aggregation did not decrease significantly, and a homogeneous distribution was not observed in samples obtained after short grinding times. In contrast, sintering at 650 °C resulted in granular structures with smaller, nanometer-scale grains and more homogeneous phase composition. Furthermore, the reduction in particle size, the formation of spherical structures, and longer grinding times contributed to a more stable observation of the material’s crystallization process.

Table 3 and Table 4 present the 2θ, FWHM, crystallite sizes (〈D〉), and microstrain (〈ε〉) values of the sintered samples of MnAlCuFeTi high-entropy alloys at sintering temperatures of 550 °C and 650 °C. The grain sizes were calculated using the Scherrer formula (Equation (6)) [28], and the obtained values were reduced to the nanoscale. At a sintering temperature of 550 °C, a grain size of 54.44 nm was obtained for the 5 h sample and 21.73 nm for the 20 h sample. At a sintering temperature of 650 °C, values of 43.97 nm for the 5 h sample and 18.36 nm for the 20 h sample were calculated. Microstrain (ε), Full Width at Half Maximum (FWHM) values, and Bragg angles (2θ) were calculated using Equation (7). At a sintering temperature of 550 °C, the microstrain was 1.70 × 10^−3^ for the 5 h sample and increased to 4.30 × 10^−3^ for the 20 h sample. At a sintering temperature of 650 °C, the microstrain value was calculated as 2.10 × 10^−3^ for the 5 h sample and 5.01 × 10^−3^ for the 20 h sample. These findings indicate that as the grinding time increases, the grain size decreases and phase stability increases. The decrease in grain size promotes the formation of nanostructures, which lead to significant increases in wear, hardness, and relative density.

However, the increased microstrain also has implications for the material’s corrosion behavior. While higher microstrain can improve mechanical properties, it can also increase the material’s susceptibility to localized corrosion. This is because the grain boundaries, where microstrain is concentrated, are more likely to act as preferential sites for corrosion, especially when the oxide layer at these boundaries is less protective. At 650 °C, the enhanced microstrain can lead to a more homogeneous structure, which can be less effective in forming a protective oxide layer, thereby accelerating corrosion. These findings highlight the balance between microstructural refinement and the potential for corrosion susceptibility. The microstrain values indicate a trade-off between improving mechanical properties and maintaining corrosion resistance, influenced by both milling time and sintering temperature. These observations underscore the importance of optimizing processing parameters to achieve the desired material performance in terms of both mechanical strength and corrosion resistance.

(6) $$D=\frac{K\lambda }{Bcos\theta \;}\;\;$$

(7) $$\varepsilon =\frac{\beta }{4tan\theta \;}\;\;$$

Here, *K* = 0.9 is the shape factor, *λ* = 0.15406 nm (Cu Kα radiation), *β* is the measured FWHM (in radians), and *θ* is the Bragg angle.

**Figure 4:**
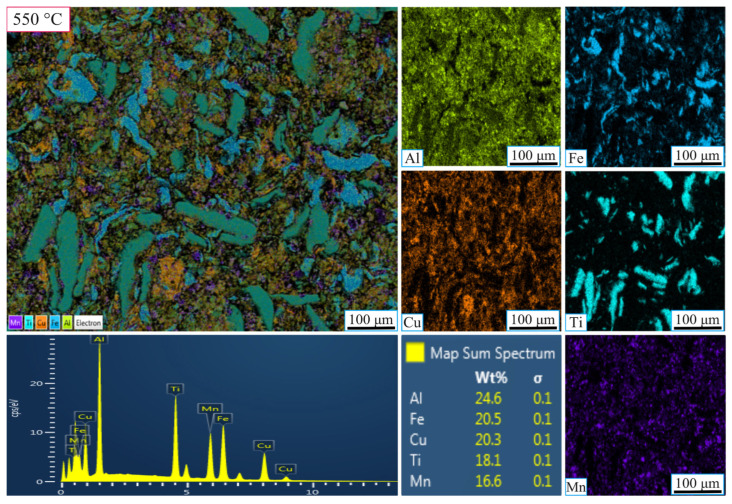
SEM-EDX micrographs showing the elemental mapping of the high-entropy MnAlCuFeTi alloy sintered at 550 °C for 5 h.

**Figure 5:**
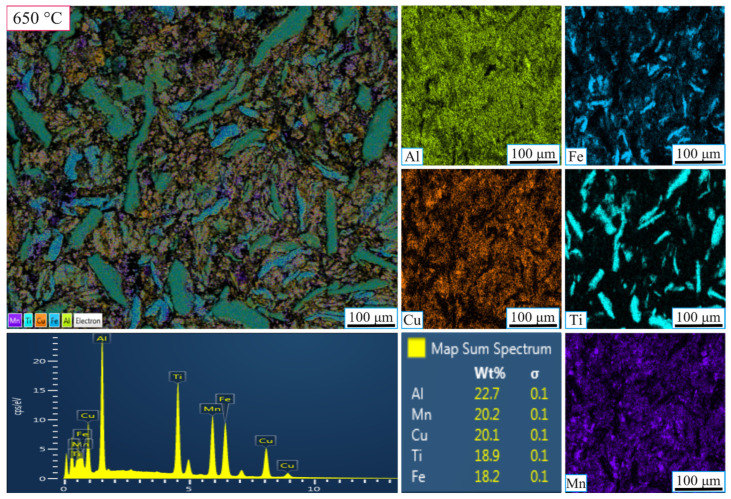
SEM-EDX micrographs showing the elemental mapping of the high-entropy MnAlCuFeTi alloy sintered at 650 °C for 5 h.

Figure 4 and Figure 5 present the SEM-EDX elemental maps of the MnAlCuFeTi high-entropy alloy sintered at 550 °C and 650 °C, with grinding times of 5 h (Figure 4 and Figure 5). In Figure 4, the sample obtained after 5 h of grinding at 550 °C exhibits a more heterogeneous elemental distribution. Al, Cu, Fe, Ti, and Mn are concentrated in certain regions, indicating that the short grinding time did not allow sufficient diffusion and phase homogenization. The structure remains non-homogeneous, and Ti forms sharp, flat-edged structures, suggesting incomplete crystallization. EDX analysis showed the following weight percentages: Al (24.6%), Fe (20.5%), Cu (20.3%), Ti (18.1%), and Mn (16.6%). While the values are close to the theoretical distribution, the overall structure lacks uniformity.

**Figure 6:**
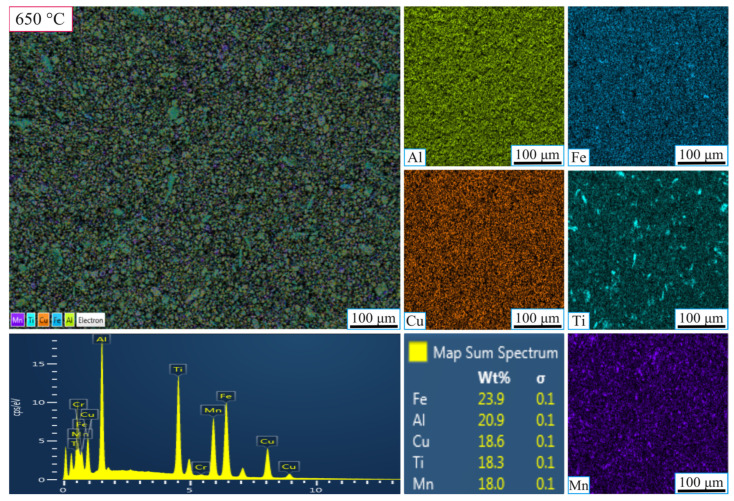
SEM-EDX micrographs showing the elemental mapping of the high-entropy MnAlCuFeTi alloy sintered at 650 °C for 20 h.

In Figure 5 (sintered at 650 °C), the sample ground for 5 h also exhibits a more heterogeneous distribution compared to the 20 h grinding time at 650 °C (Figure 6). However, there is a slight improvement in the uniformity of elemental distribution. Fe and Ti form denser clusters, but overall elemental diffusion remains insufficient and phase stability incomplete. EDX analysis revealed the following weight percentages: Al (22.7%), Fe (18.2%), Cu (20.1%), Ti (18.9%), and Mn (20.2%), which are close to theoretical values, but the structure is still not homogeneous, suggesting that 5 h of grinding is not sufficient for full diffusion and homogenization at this higher sintering temperature.

**Figure 7:**
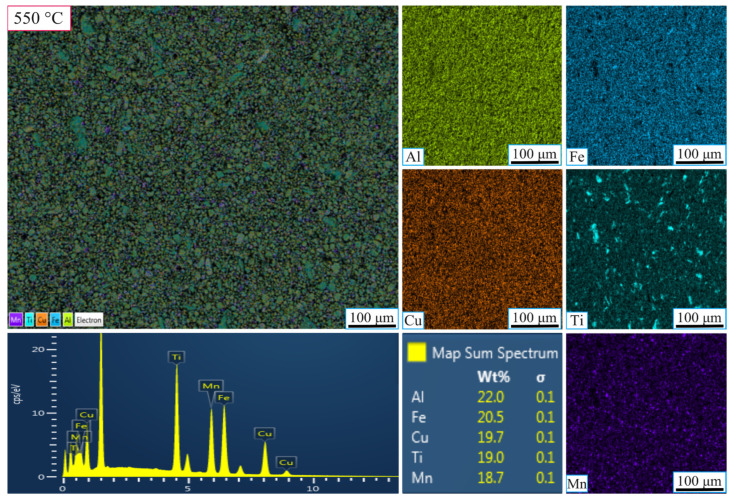
SEM-EDX micrographs showing the elemental mapping of the high-entropy MnAlCuFeTi alloy sintered at 550 °C for 20 h.

Figure 6 and Figure 7 show the results for samples ground for 20 h at 550 °C and 650 °C, respectively. In Figure 7 (at 550 °C), the 20 h grinding period resulted in a more homogeneous distribution of Al, Cu, Fe, Ti, and Mn, as well as a finer-grained structure. The extended grinding time enhanced cold-induced tearing and fracture, improving element diffusion and crystallization. EDX analysis showed the following weight percentages: Al (22%), Fe (20.5%), Cu (19.7%), Ti (19%), and Mn (18.7%), confirming that longer grinding times lead to a more uniform and stable microstructure, with the formation of nanometer-sized particles.

In Figure 6 (at 650 °C), the 20 h grinding time resulted in a more homogeneous elemental distribution and a finer-grained structure. The higher sintering temperature combined with the extended grinding period promoted better diffusion of the elements, improving phase stability and resulting in a more balanced distribution of Al, Cu, Fe, Ti, and Mn. EDX analysis revealed the following weight percentages: Fe (23.9%), Al (20.9%), Cu (18.6%), Ti (18.3%), and Mn (18%). This confirms that both the longer grinding time and higher sintering temperature contributed to a more homogeneous and stable microstructure.

As a result, at 550 °C, the sample obtained after 5 h of grinding exhibited a heterogeneous elemental distribution and insufficient homogenization. However, with 20 h of grinding, the structure became more uniform, with a finer grain size and increased phase stability. In contrast, at 650 °C, the 5 h grinding sample showed a more uniform elemental distribution than at 550 °C, but phase stability was still not fully achieved. After 20 h of grinding at 650 °C, the elements were more evenly distributed, and the microstructure was significantly finer and more stable. The higher sintering temperature facilitated improved diffusion, enhancing the material’s homogeneity and overall stability.

### 3.3. Density and Microhardness Measurements

**Figure 8:**
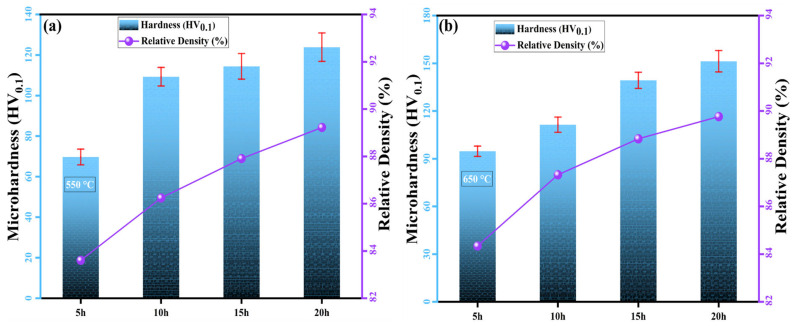
The relationship between microhardness and relative density of the high-entropy MnAlCuFeTi alloy as a function of mechanical alloying time for samples sintered at (**a**) 550 °C and (**b**) 650 °C.

Figure 8 depicts the change in microhardness and relative density values of the sintered MnAlCuFeTi high-entropy alloy as a function of grinding time at sintering temperatures of 550 °C and 650 °C. For the samples produced with a 5 h alloying time and sintered at 550 °C, the microhardness was approximately 78 HV, and the relative density was 83.6%. These values indicate that the short grinding time resulted in large grains and that phase stability was not fully achieved, thus diffusion was insufficient. SEM images revealed that the elements were homogeneously distributed and the microstructure was heterogeneous. Grinding time characteristics, particularly at 10, 15, and 20 h, showed increases in microhardness and relative distribution values, respectively. Over a 20 h alloying period, microhardness increased to 85 HV, and relative distribution rose to 89.2%. These increases confirm the enhanced phase stability and reduced particle breakage, as indicated by XRD analyses.

Sintering at 650 °C resulted in a microhardness of 94.7 HV and a relative density of 84.3% for the samples ground for 5 h. The elevated sintering temperature was found to improve elemental homogeneity by accelerating diffusion, although the grain size did not decrease significantly due to the shorter grinding time. However, at grinding times of 10, 15, and 20 h, both microhardness and relative density showed distinct variations, reflecting the effects of prolonged grinding on the material’s properties. After 20 h of grinding, the microhardness increased to 151.3 HV, and the relative density reached 89.8%. These results suggest that higher sintering temperatures and longer grinding times enhance diffusion, reduce grain size, improve stability, and promote the formation of more homogeneous structures. Consistent with XRD and SEM analyses, these improvements increase yield and dispersion, leading to a more stable material structure.

In summary, the effects of grinding time and sintering temperature on the microstructure, as well as the relative densities at sintering temperatures of 550 °C and 650 °C, have been thoroughly examined. Additionally, in conjunction with SEM and XRD analyses, significant improvements in yield and distribution, including particle shrinkage, changes in phase stability, and homogenization of micro-distribution, are consistent with these results. These findings provide insights into how grinding time and sintering temperature influence the material’s mechanical and physical properties.

**Figure 9:**
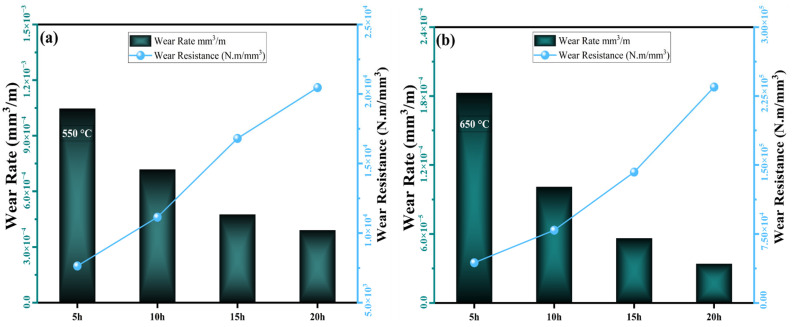
Variation in wear rate and wear resistance of the high-entropy MnAlCuFeTi alloy with mechanical alloying time for samples sintered at (**a**) 550 °C and (**b**) 650 °C.

Wear rate and wear resistance data of the sintered MnAlCuFeTi high-entropy alloy as a function of grinding time at sintering temperatures of 550 °C and 650 °C are shown in Figure 9. At a sintering temperature of 550 °C, the sample ground for 5 h exhibited the lowest wear rate (1.05 × 10^−3^ mm^3^/m). The wear resistance, however, was at its lowest level at 7638.8 N.m/mm. This indicates that the short grinding time did not sufficiently develop phase homogeneity and diffusion, resulting in a high wear rate. As the grinding time increased, the wear rate decreased while the wear resistance increased. After 20 h of grinding, the wear rate decreased to 3.91 × 10^−4^ mm^3^/m, while the wear resistance increased to 20,466.6 N.m/mm. This shows that as grinding time increases, wear resistance increases and the wear rate decreases, due to reduced particle size and improved phase homogeneity.

At a sintering temperature of 650 °C, the wear rate decreased to 1.83 × 10^−4^ mm^3^/m, while the wear resistance increased to 43,739.8 N.m/mm in the sample obtained after 5 h of grinding. The high sintering temperature increased phase homogeneity by accelerating diffusion and improved wear resistance. At grinding times of 10, 15, and 20 h, the wear rate decreased further, while wear resistance increased significantly. At the end of the 20 h grinding period, the wear rate decreased to 3.41 × 10^−5^ mm^3^/m, while the wear resistance increased to 234,879.6 N.m/mm. These data support the finding that the 650 °C sintering temperature accelerates diffusion, thereby increasing phase stability and homogeneity, which in turn elevates wear resistance and reduces the wear rate.

In conclusion, at both sintering temperatures, the wear rate decreased, and the wear resistance increased with increasing grinding time. However, at the 650 °C sintering temperature, the high temperature and prolonged grinding time accelerated diffusion and improved phase homogeneity, thereby raising wear resistance to levels exceeding the maximum observed at 550 °C. A grinding time of 20 h yielded the lowest wear rate and highest wear resistance at both sintering temperatures. This demonstrates that grain refinement and improved phase stability significantly enhance wear properties.

**Figure 10:**
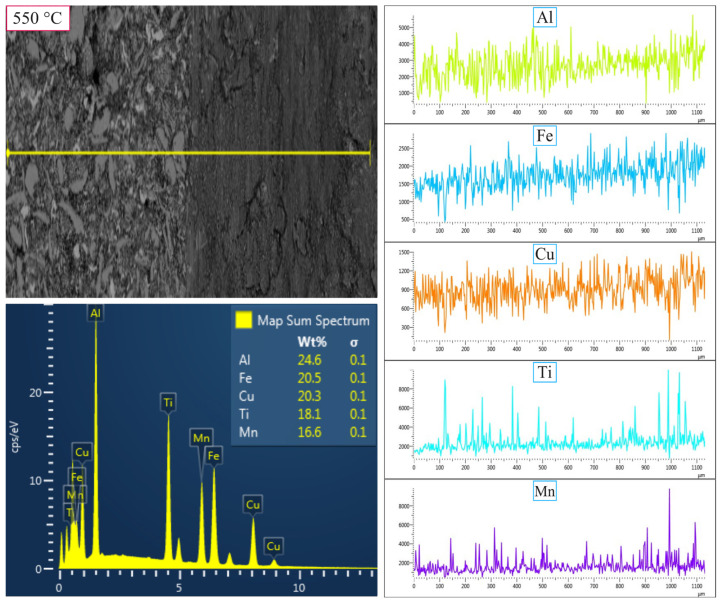
SEM micrographs and EDS line-scan analysis of the worn surfaces of the high-entropy MnAlCuFeTi alloy, as influenced by mechanical alloying time, for samples sintered at 550 °C for 5 h.

**Figure 11:**
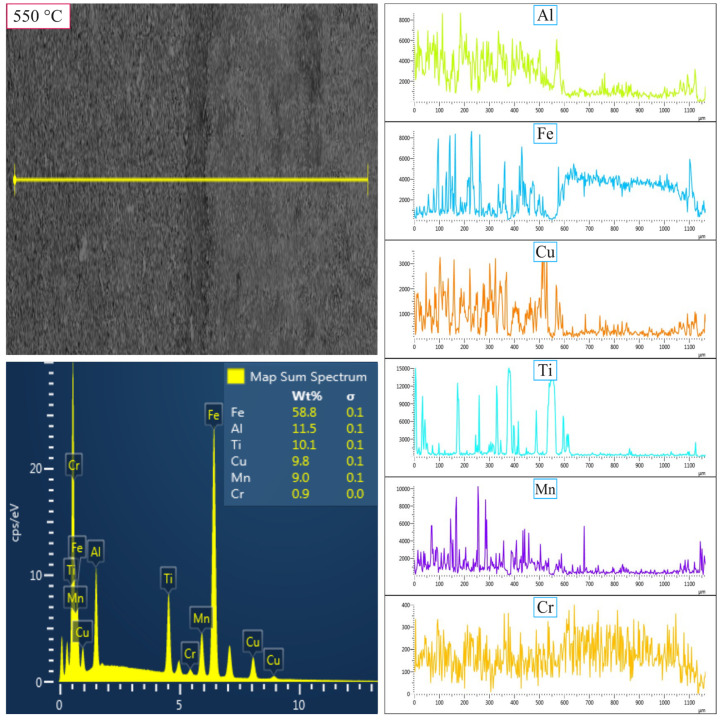
SEM micrographs and EDS line-scan analysis of the worn surfaces of the high-entropy MnAlCuFeTi alloy, as influenced by mechanical alloying time, for samples sintered at 550 °C for 20 h.

The worn surfaces of the MnAlCuFeTi high-entropy alloy sintered at 550 °C after 5 h (Figure 10) and 20 h (Figure 11) of grinding, along with elemental variation analyses performed using the Linescan method, are shown in Figure 10 and Figure 11. In the sample obtained with a 5 h grinding time, the elements are homogeneously distributed on the unabraded surface, and there is no clear indication of an increase in Fe and Cr elements on the abraded surface. In this case, the abrasive steel balls easily abraded the sample, and no material transfer from the balls or ball wear was detected. This indicates that the particle size is large and that phase stability is insufficiently developed, resulting in non-homogeneous surface wear. In the sample obtained after a 20 h grinding period, a noticeable change was observed on the worn surface. Linescan analysis revealed a significant increase in Fe and Cr elements on the worn surface. Specifically, the amount of Fe increased on the worn surface, and Cr was also detected. This situation shows that while the abrasive steel ball wore down the 20 h sample, which has high wear resistance, the ball itself also wore down, and Fe and Cr elements were transferred from the ball to the surface. According to the EDS Linescan data, this material transfer has been detected, and the increase in the Fe-Cr ratio clearly shows that ball wear leads to accumulation on the worn surface.

**Figure 12:**
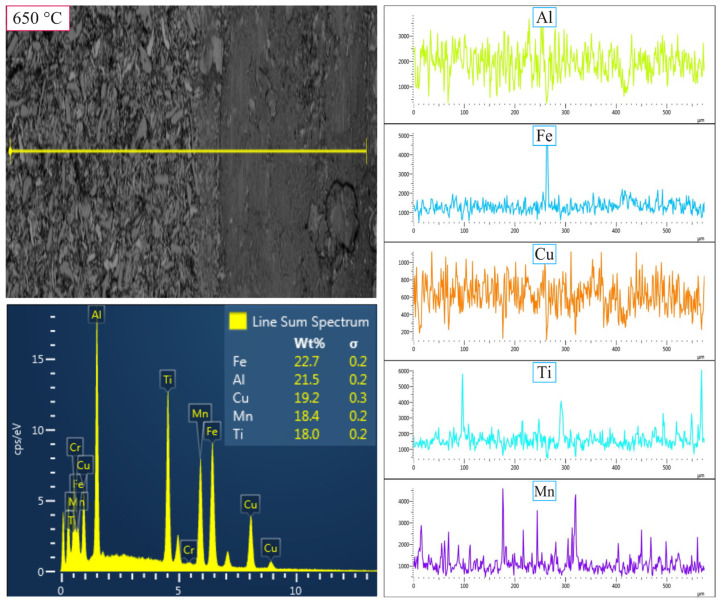
SEM micrographs and EDS line-scan analysis of the worn surfaces of the high-entropy MnAlCuFeTi alloy, as influenced by mechanical alloying time, for samples sintered at 650 °C for 5 h.

**Figure 13:**
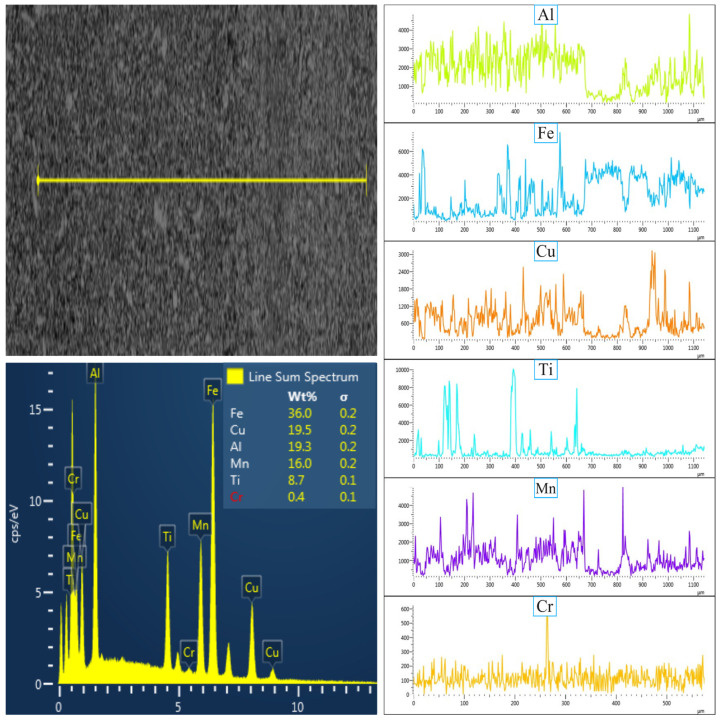
SEM micrographs and EDS line-scan analysis of the worn surfaces of the high-entropy MnAlCuFeTi alloy, as influenced by mechanical alloying time, for samples sintered at 650 °C for 20 h.

Figure 12 and Figure 13 show the worn surfaces of the MnAlCuFeTi high-entropy alloy sintered at 650 °C after 5 h (Figure 12) and 20 h (Figure 13) of grinding, along with elemental variation analyses performed using the Linescan method. In the sample obtained with a 5 h grinding time, the elements are distributed homogeneously on the unabraded surface, and an increase in the Fe ratio is observed on the abraded surface, while no Cr element was detected. This indicates that material loss occurred largely without ball wear, due to the force exerted by the abrasive steel ball on the material surface, and that no elemental transfer occurred from the ball. The reason for this is the large grain size and insufficient phase stability, which led to non-homogeneous surface wear.

In the sample obtained after 20 h of grinding, the elemental changes on the worn surface are much more pronounced. Linescan analysis clearly shows an increase in the Fe ratio and the presence of Cr on the worn surface. In particular, the Fe content has increased on the worn surface, and Cr has also been detected. This situation shows that while the abrasive steel ball wears down the 20 h sample, which has high wear resistance, the ball itself also wears down, and Fe and Cr elements are transferred from the ball to the surface. According to the EDS Linescan data, the increase of these elements on the surface is an indication of ball wear and the transfer of elements from the ball to the surface.

In summary, Figure 10, Figure 11, Figure 12 and Figure 13 provide important information on elemental changes on the worn surfaces of samples ground for 5 h and 20 h, as well as on ball wear and transfer to the material surface. In the 5 h samples, the wear rate was high, whereas in the 20 h samples, the abrasive ball wore down, and Fe and Cr elements were transferred to the surface. With increasing mechanical alloying time, the compaction of powders to the nanometer level, as evidenced by wear, hardness, density, and SEM/XRD results, leads to improved mechanical properties and increased phase stability.

### 3.4. Electrochemical Corrosion Test

**Figure 14:**
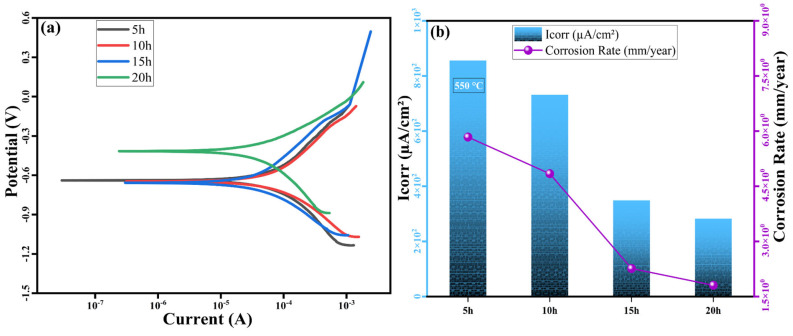
(**a**) Polarization curves, (**b**) trend of *I_corr_* variation, and the change in corrosion rate for the high-entropy MnAlCuFeTi alloy sintered at 550 °C.

**Figure 15:**
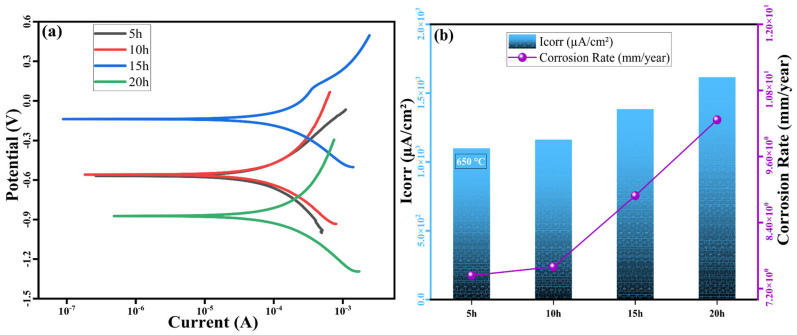
(**a**) Polarization curves, (**b**) trend of *I_corr_* variation, and the change in corrosion rate for the high-entropy MnAlCuFeTi alloy sintered at 650 °C.

Figure 14 and Figure 15 display the polarization curves (a) and changes in current density (*I_corr_*) as well as the corrosive wear rate (b) for the MnAlCuFeTi high-entropy alloy samples sintered at 550 °C and 650 °C. Additionally, Table 5 and Table 6 present the corresponding polarization results for the two sintering temperatures. In Figure 14a, the polarization curve for the sample ground for 5 h shows the highest current density (*I_corr_*) of 855.78 µA/cm^2^, indicating greater susceptibility to corrosion and relatively lower corrosion resistance. As the grinding time increases, a noticeable decline in *I_corr_* is observed. At 10 h, the value decreases to 732.10 µA/cm^2^, then drops to 348.94 µA/cm^2^ after 15 h, and reaches 282.63 µA/cm^2^ after 20 h. These findings suggest that as particle size decreases with longer milling times, the material’s corrosion resistance improves significantly. The trend observed in the corrosion wear rate data, presented in Table 5, further supports this. For the 5 h milled sample, the wear rate is 5.83 mm/year, whereas after 20 h of grinding, it decreases significantly to 1.80 mm/year, confirming the formation of a more compact and corrosion-resistant microstructure with extended grinding [29].

Turning to Figure 15a, which shows the polarization curves for samples sintered at 650 °C, we observe that the sample ground for 5 h exhibits the highest *I_corr_* value of 1100 µA/cm^2^. Correspondingly, the corrosive wear rate recorded in Table 6 is 7.43 mm/year. As the grinding time increases, the *I_corr_* values rise, reaching 1163.15 µA/cm^2^, 1384.21 µA/cm^2^, and 1615.78 µA/cm^2^ at 10, 15, and 20 h, respectively. This pattern suggests that the 650 °C sintering temperature accelerates diffusion within the material, resulting in greater phase homogeneity but also weakening its corrosion resistance. Similarly, the corrosive wear rate increases from 7.43 mm/year at 5 h of grinding to 10.26 mm/year at 20 h of grinding, indicating that high-temperature sintering at 650 °C adversely affects the alloy’s corrosion properties. High sintering temperatures promote phase homogeneity by accelerating internal diffusion processes within the material. However, this increase in homogeneity can reduce corrosion resistance. At 650 °C, the formation of intermetallic phases in the microstructure becomes more pronounced, potentially creating surfaces that are more prone to corrosion [30,31]. Additionally, the enhanced homogeneity of the material can lead to faster corrosion, as a more uniform surface may prevent the formation of a protective oxide layer, which typically provides corrosion resistance [32,33,34]. These results indicate that while both sintering temperature and grinding time play key roles in optimizing the alloy’s properties, the high sintering temperature of 650 °C adversely affects corrosion resistance despite improvements in phase structure and homogeneity.

## 4. Conclusions

In this study, the effects of mechanical alloying time and sintering temperature on the microstructure, mechanical properties, and corrosion behavior of MnAlCuFeTi high-entropy alloys were investigated. Increasing the mechanical alloying time led to a finer grain size, promoting the formation of a nanostructure. Calculations using the Scherrer formula indicate that the crystallite size was 54.44 nm after 5 h of grinding and decreased to 21.73 nm after 20 h of grinding. The SEM images and XRD analyses confirm this reduction and the improvement in phase homogeneity as milling time increases.

The samples ground for 20 h showed the highest hardness (123.87 HV) and density (7.01 g/cm^3^) values in the hardness and density tests. SEM-EDX line-scan analysis shows that elements are homogeneously distributed on the surface after 5 h of grinding, whereas after 20 h of grinding, Fe and Cr increase on the worn surface, and contamination from the ball is transferred to the surface. This confirms the effect of ball wear.

At a sintering temperature of 550 °C, the *I_corr_* value decreased from 855.78 µA/cm^2^ to 282.63 µA/cm^2^, while the corrosive wear rate decreased from 5.83 mm/year to 1.80 mm/year. This indicates that corrosion resistance increases with decreasing grain size. At a sintering temperature of 650 °C, the *I_corr_* value increased from 1100 µA/cm^2^ to 1615.78 µA/cm^2^, and the corrosive wear rate increased from 7.43 mm/year to 10.26 mm/year. This indicates that corrosion resistance weakens as phase homogeneity improves.

In conclusion, sintering temperature and grinding time are critical parameters for optimizing the mechanical and corrosion properties of MnAlCuFeTi high-entropy alloys. At a sintering temperature of 550 °C, grinding time improves corrosion resistance and mechanical properties, whereas at 650 °C, high-temperature phase homogeneity negatively affects corrosion properties. These findings indicate that determining the optimal sintering and grinding parameters for HEAs is necessary for high-performance applications.

## Figures and Tables

**Table 1:** Theoretical, experimental, and relative densities of the high-entropy MnAlCuFeTi alloy sintered at 550 °C.

Materials Code	Theoretical Density (g/cm^3^)	Experimental Density (g/cm^3^)	Relative Density (%)
**5 h**	5.16	4.314	83.604
**10 h**	5.16	4.45	86.240
**15 h**	5.16	4.536	87.906
**20 h**	5.16	4.604	89.224

**Table 2:** Theoretical, experimental, and relative densities of the high-entropy MnAlCuFeTi alloy sintered at 650 °C.

Materials Code	Theoretical Density (g/cm^3^)	Experimental Density (g/cm^3^)	Relative Density (%)
**5 h**	5.16	4.352	84.341
**10 h**	5.16	4.506	87.325
**15 h**	5.16	4.584	88.837
**20 h**	5.16	4.632	89.767

**Table 3:** Crystallite Size (〈D〉) and Microstrain (〈ε〉) of the high-entropy MnAlCuFeTi alloy sintered at 550 °C.

	2θ (°)	FWHM (°)	Crystallite Size 〈D〉 (nm)	Microstrain 〈ε〉 (×10^−3^)
**5 h**	44.04	0.157	54.44	1.70
**10 h**	44.30	0.196	43.58	2.11
**15 h**	43.93	0.295	32.65	3.19
**20 h**	43.55	0.393	21.73	4.30

**Table 4:** Crystallite Size (〈D〉) and Microstrain (〈ε〉) of the high-entropy MnAlCuFeTi alloy sintered at 650 °C.

	2θ (°)	FWHM (°)	Crystallite Size 〈D〉 (nm)	Microstrain 〈ε〉 (×10^−3^)
**5 h**	44.20	0.195	43.97	2.10
**10 h**	44.24	0.307	27.93	3.30
**15 h**	43.54	0.387	22.10	4.23
**20 h**	44.14	0.467	18.36	5.01

**Table 5:** Results of polarization testing of the 550 °C sintered MnAlCuFeTi high-entropy alloy.

Materials Code	E_corr_ (mV)	*I_corr_* (µA/cm^2^)	Corrosion Rate (mm/year)
**5 h**	−639	855.78	5.83
**10 h**	−647	732.10	4.84
**15 h**	−658	348.94	2.26
**20 h**	−416	282.63	1.80

**Table 6:** Results of polarization testing of the 650 °C sintered MnAlCuFeTi high-entropy alloy.

Materials Code	E_corr_ (mV)	*I_corr_* (µA/cm^2^)	Corrosion Rate (mm/year)
**5 h**	−569	1100	7.43
**10 h**	−559	1163.15	7.59
**15 h**	−138	1384.21	8.88
**20 h**	−873	1615.78	10.26

## Data Availability

The original contributions presented in this study are included in the article. The raw data supportng the conclusions of this article are available upon request from the corresponding author.
